# Studies needed to address public health challenges of the 2009 H1N1 influenza pandemic: insights from modeling

**DOI:** 10.1371/currents.RRN1135

**Published:** 2009-12-17

**Authors:** The WHO Informal Network for Mathematical Modelling for Pandemic Influenza H1N1 2009 (Working Group on Data Needs), Maria D Van Kerkhove, Tommi Asikainen, Niels Becker, Steven Bjorge, Jean-Claude Desenclos, Thais dos Santos, Christophe Fraser, Gabriel M Leung, Marc Lipstich, Ira M Longini, Emma McBryde, Cathy Roth, David K Shay, Derek Smith, Jacco Wallinga, Peter White, Neil M Ferguson, Steven Riley

**Affiliations:** MRC Centre for Outbreak Analysis and Modelling, Imperial College London, UK; European Centre for Disease Prevention and Control, Stockholm, Sweden; National Centre for Epidemiology and Population Health, The Australian National University, Australia; World Health Organization; Institut de Veille Sanitaire, France; World Health Organization; MRC Centre for Outbreak Analysis and Modelling, Imperial College London, UK; Food and Health Bureau, Government of the Hong Kong SAR; Department of Epidemiology, Harvard University, USA; Center Stat. and Quant. Infect Dis (CSQUID), Vaccine and Infectious Diseases Institute. Hutchinson Research Center, US and the Department of Biostatistics, University of Washington, School of Public Health; Victorian Infectious Diseases Service, Royal Melbourne Hospital, Australia; World Health Organization; Influenza Division, US Centers for Disease Control and Prevention, Atlanta, USA; Department of Zoology, University of Cambridge, UK; Erasmus Medical Center, Department of Virology, Rotterdam, The Netherlands; and Fogarty International Center, National Institutes of Health, Bethesda, MD, USA; National Institute of Public Health and the Environment (RIVM), Centre for Infectious Disease Control Netherlands and the University Medical Center Utrecht, Division Julius Center for Health Sciences and Primary Care, The Netherlands; MRC Centre for Outbreak Analysis and Modelling, Imperial College London, UK and the Modelling and Economics Unit, Health Protection Agency Centre for Infections, UK; MRC Centre for Outbreak Analysis and Modelling, Imperial College London, UK; School of Public Health and Department of Community Medicine, The University of Hong Kong, Hong Kong SAR

## Abstract

The 2009 influenza pandemic (H1N1pdm) has completed its first wave in many northern and southern hemisphere populations and many northern hemisphere populations are reporting substantial activity indicating the start of a second wave this autumn. As the global epidemiology of this novel strain unfolds, substantial policy challenges will continue to present themselves for the next 12 to 18 months. Here, we anticipate six public health challenges and identify data that are required for public health decision making. In particular, we suggest studies that will generate data not otherwise available from routine surveillance. Representative serological surveys stand out as a critical source of data with which to reduce uncertainty around policy choices for both pharmaceutical and non-pharmaceutical interventions after the initial wave has passed. Also, monitoring the time course of incidence of severe H1N1pdm cases will give a clear picture of variability in underlying transmissibility of the virus during population wide changes in behavior such as school vacations and other non-pharmaceutical interventions. In addition, we address low resource settings where routine surveillance for influenza has not been established and suggest alternative ways to collect data for the 2009 (and beyond) influenza H1N1 pandemic.

## Introduction

The emergence and global spread of a novel strain of human influenza A H1N1 (H1N1pdm) during 2009 has highlighted the importance of data from both detailed outbreak investigation and population surveillance for the support of public health decision making. For example, public health organizations in several countries undertook detailed case investigations to build databases of the first few hundred cases, which include laboratory confirmation status, age, relative severity, exposure history, onset of symptoms, and contact history, e.g. the UK First Few Hundred project [1]. Descriptive analyses of such data allowed decision-makers to conclude rapidly that the disease caused by novel strain was relatively mild for the majority of confirmed cases and that it was being transmitted efficiently between children. Most countries therefore decided that stringent interventions at the community level (such as proactive school closures) were not appropriate because their benefits were limited when compared with the high overall cost to society. Population surveillance was also crucial in the early stages of the pandemic. Indeed, the two independent influenza cases [2] that provided the viral isolates used to discern the presence of a novel strain were obtained through a sentinel surveillance system designed for exactly that purpose[3]. 

In recent years, it has become common to use mathematical modeling to analyze the underlying disease dynamics of outbreaks. Parameters such as the reproductive number *R* [4], which can be estimated from outbreak investigation data [5], give insight into how underlying transmission dynamics will influence the likely impact of possible interventions. For example, if the underlying basic reproductive number, *R*_0_, is low, the impact of community-based mitigation strategies against a severe influenza pandemic might be substantial [6] . However, the use of specific mathematical models to explicitly support particular policy decisions masks a more general aspect of decision making, namely, the inclusion of ‘modelers’ in the policy advice process to ensure that quantitative insights into epidemic dynamics are available. This article is the result of the first meeting of an informal network convened by the World Health Organization (WHO) for the modeling of pandemic H1N1 influenza. The network is made up of public health professionals, policy makers and scientists with expertise in the transmission dynamics, epidemiology, ecology and evolution of human infectious diseases [7].

The H1N1pdm pandemic will continue to generate novel challenges for public health decision makers over the next 1-2 years.  In this article, we suggest likely challenges and consider how uncertainties over the disease dynamics may affect policy formulation. The main objective of this exercise is not to provide evidence to support specific policy alternatives. Rather, we try to anticipate and prioritize non-routine data that should be planned and funded in the short term to be of significant value to policy makers in the medium and longer term.

## Public health challenges

### Measuring age-specific immunity to infection.

**Figure 1.**
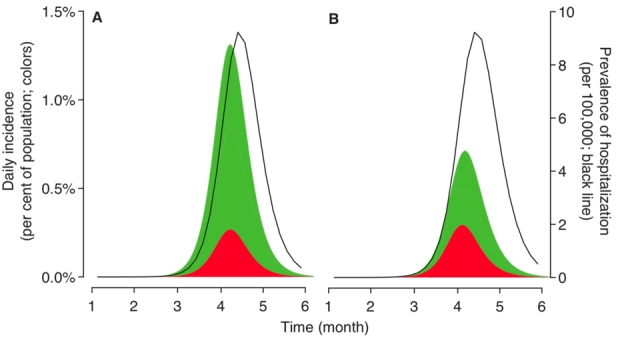
Epidemic curves based on surveillance data could mask quite different underlying transmission dynamics. We used deterministic SIR model [25] with two age classes: children (20% of the population) and adults (80%). The initial doubling time was set to 5 days with a 2.6 day generation time. These parameters imply a basic reproductive number of 1.4 (for this model [5]). The seed was equivalent to one infectious individual in a population of 7 million at time 0 and mixing between age groups was consistent with contact diary data for the UK [26] (children defined as <20 years). The shaded regions show daily incidence of symptomatic cases for children (red) and adults (green). We assumed 86% of infections were symptomatic [8] . The black line is the estimated number of hospital beds required at a given time. The susceptibility of children relative to adults was parameterized using the ratio of child cases to adult cases during the exponential phase of epidemic growth. **A **shows a baseline scenario. The ratio of early cases was proportionate to the population (20:80, children:adults) and all ages were equally likely to require hospitalization. **B** shows a scenario likely to be closer to current nH1N1 dynamics. The ratio of early cases was 50:50 and adults were much more likely to require hospitalization.

To be able to estimate the susceptibility of a population to future similar strains, it is important to understand the reason that initial epidemics of a new strain fade out. Using routinely collected data, it will be difficult to know with confidence why any particular local epidemic of the H1N1pdm strain ends (Figure 1). It may be that the number of susceptible individuals has been depleted by the development of immunity, that a population-wide public health response to the epidemic has occurred (and was sustained), or that transmissibility dropped for seasonal reasons. Most likely, local fade-outs will be due to a combination of these factors. Routinely collected data such as influenza-like-illness (ILI) reporting from sentinel networks of physicians and reported hospitalizations suffer from the usual caveats of discounting a potentially large unobserved subclinical population, the uncertain contextual heterogeneities of health care seeking patterns, and difficult etiologic interpretation in locations where seasonal influenza strains, or indeed other upper respiratory viruses with similar presentations, co-circulate with H1N1pdm. Representative serological surveys provide the only viable means to infer population-level susceptibility with any accuracy, especially if there is a substantial proportion of an asymptomatic infection.

Currently available data suggest that children are more likely to become infected than adults [8] [9] . With a relatively low overall transmissibility, these characteristics are likely to lead to a lower attack rate among adults and a higher attack rate among children. Therefore, one way in which H1N1pdm could evolve to improve its transmissibility at the end of an initial wave of infection would be to improve its ability to infect adults, with or without a substantial antigenic change. For instance, a shift in the transmission efficiency of the virus between droplet and aerosol could affect children and adults in different ways. Should H1N1pdm evolve in this way, knowing, with confidence the proportion of the population exposed during the initial wave will be of substantial public health value. Such changes would be especially important given that at present, the severity of illness for confirmed cases seems to be higher in adults than in children [9] [10].

### Accurately quantifying severity. 

Accurate estimates of the per-person risk of severe outcomes, such as the case hospitalization ratio (i.e., the number of hospitalized cases divided by total number of infections), the hospitalization ICU ratio (the total number of cases requiring intensive care divided by total number of hospitalized cases) and the case-fatality ratio (the total number of deaths that are caused by H1N1pdm infection divided by the total number of infections), are required for planning purposes and also to provide at-risk individuals with the best possible information. Unfortunately, reporting biases for both the numerator and the denominator in hospitalization ratio calculations make accurate estimates difficult. For example, mild infections in young children are much more likely to be reported than mild infections in adults, whereas deaths attributable to H1N1pdm will depend on testing capacity and policy.  In addition, some countries have hospitalized cases for isolation purposes, rather than because individuals were suffering severe illness. Therefore, quantifying the overall exposure of the population using a time series of representative, age-stratified serological surveys will greatly improve the accuracy of our estimates of risk, by giving definitive denominator information.

### Improving treatment outcomes for severe cases.

Although not directly linked to epidemic dynamics, we suggest that hospital-based cohort studies of H1N1pdm cases are needed to assess the pathogenicity of H1N1pdm infection.  These studies should collect detailed information on the clinical spectrum of disease including onset and duration of symptoms, prevalence of underlying conditions (such as pregnancy, chronic respiratory disease, immunosuppression, smoking, obesity, chronic respiratory conditions, diabetes and neurologic disorders), duration of hospital/ICU stay, complications from infection including bacterial super-infection, antiviral/antibiotic treatment including when administered, the efficacy of other adjunctive measures (such as immune modulation, novel oxygenation or ventilation strategies, etc) and serial blood and respiratory samples for RT-PCR and virus culture to determine the extent and duration of viral shedding and antiviral treatment failure.  The WHO, US Centers for Disease Control and Prevention Canadian Clinical Trials Group, South East Asian Infectious Disease Clinical Research Network  have developed clinical data collection forms for such studies[11] [12], which could be adapted to be context specific and implemented in a representative group of hospitals in each participating country. Although these studies will not capture the mild spectrum of illness (as discussed above), they will fill the current data gaps about pathogenicity and the clinical course of illness including prognostic information. The use of propensity scores could yield valuable insights into the relative efficacy of different treatment strategies in the short term, while awaiting results of prospective trials.

### Quantifying the efficacy of interventions.

After an initial establishment phase, changes in the growth rate of a novel infectious disease can provide an accurate measure of changes in transmission rates. If the doubling time of the number of new cases is constant in the early stages, then significant changes in underlying transmissibility are unlikely to have occurred. However, if the doubling time appears to slow down during school vacations/holidays/closures or during other widespread changes in mixing, then this likely indicates a genuine shift in the rate of disease transmission. Accurate measures of changes in the growth rate – and possibly also the age-composition of reported cases – are required to quantify the population-wide effect of changes in behavior, such as the start of school vacations and restrictions in mass gatherings. Countries with hospital based respiratory surveillance systems, which are often not optimized in its data specification and collection, could be enhanced to collect more detailed clinical and laboratory data (described above) from ILI, ARI (acute respiratory illness) and SARI (severe acute respiratory illness) patients [13]. In addition, clinical information of ILI, ARI and SARI patients paired with laboratory testing could provide estimates of the burden of seasonal influenza compared with that of pandemic influenza.

### Capturing the full impact of the pandemic on mortality.

We should aim to monitor excess mortality due to H1N1pdm in the timeliest way possible. The number of deaths attributable to seasonal and previous pandemic influenza is considerably higher than the number certified by vital statistics registration as due to influenza or by the number of influenza deaths reported through surveillance schemes [14]; the total number estimated depends strongly on whether the excess above baseline is confined to deaths from pneumonia and influenza, or whether all respiratory and circulatory deaths or all cause deaths are considered [15]. Often, a number of causes contribute to individual mortality. Influenza-associated mortality has traditionally been estimated as the excess pneumonia and influenza (P & I) mortality above a baseline of deaths during seasonal influenza epidemic periods. Excess P & I mortality estimates are often not timely, as data compilation can take months. To monitor influenza excess mortality in a more timely fashion, several countries have set up sentinel systems that they integrate in their routine influenza surveillance (e.g., European monitoring of excess mortality for public health action (EuroMoMo) www.euromomo.eu). The US CDC established a sentinel system in 122 US cities several decades ago. More recently, several European countries have developed real time monitoring schemes of mortality in which number of deaths by age are transmitted electronically from all or a subset of municipalities to a central database. These schemes allow much more rapid assessment of overall mortality trends and should be utilized in near-real time during the upcoming Northern Hemisphere influenza season to ensure that policy makers are continuously kept abreast of how excess mortality for the pandemic will compare with similar statistics often quoted for seasonal influenza.

### Rapidly identifying and responding to antigenic variants.

It will be useful to isolate virus from infected individuals for whom there is also a serum sample. Although doing this systematically from all cases would place an intolerable burden on supporting laboratory services, there will be value in developing sub-studies amongst larger serological surveys. Obtaining such paired virological and serological data from vaccinated individuals will be particularly useful because it will allow the investigation of antiviral resistance and vaccine failure.  In some instances, vaccine failure could be due to an antigenic variant that is not protected by immunity raised against the vaccine strain.  Therefore, active sampling of symptomatic vaccinated individuals could help to provide early warning of vaccine-escape mutants, which, if they are rare initially, might take longer to be detected by routine surveillance.

## Meeting the challenges

While there have been recommendations focusing on how to maintain and enhance population-level surveillance when in most countries case numbers have far exceeded routine testing capacity [11] [16]; here we suggest specific, non-routine data that will help public health policy makers to address six public health challenges that we anticipate will continue for the next 12 to 18 months. Because of inherent biases in the routine reporting of cases of differing levels of severity, sufficiently-powered representative serological surveys will be useful in the short term and the medium term to help quantify the degree of susceptibility in the population and to help characterize individual level severity. Systematic reporting of the incidence of ARI, and SARI will help to characterize the speed of growth of the epidemic and hence allow the detection of significant changes in underlying transmissibility. Specific data gathering processes are also required to accurately define the clinical spectrum of severe disease, measure excess mortality in a timely fashion and help to rapidly detect possible vaccine escape, antiviral resistant and other mutant strains.

The need for rapid serological studies stands out among these impending knowledge gaps. Historically, the best information on circulating seasonal influenza has come from prospective community studies based on households.   Two important such studies in the US were the Tecumseh community study [17] [18] and the Seattle virus watch [19]. Study participants provided periodic serological samples every four to six months over several years.  Bracketing sera were used to detect infections through significant antibody titer rises.  In addition, the serologic data provided estimates of the degree of partial immunity in the populations under study at any point in time.  Finally, influenza symptom data coupled with virological identification provided valuable information of infection and illness attack rates by age and other demographic characteristics, as well as the pathogenicity and virulence of the identified circulating strains of influenza.  Even today, these studies provide the most complete description of the epidemiology of influenza circulating in the community. It is therefore encouraging that such detailed prospective community serologic studies are underway or planned in the many countries including Argentina, Australia, Bangladesh, Canada, Chile, France, Finland, Germany, Hong Kong, Italy, Japan, Mexico, the Philippines, Singapore, Sweden, Thailand, UK, and the US.

Observing the serological attack rate across countries gives us a standardized measure of the risk of infection across countries. Such a standardized measure facilitates international comparisons that are essential assess the effectiveness of different interventions against influenza in different countries. It is difficult to compare doctor consultations, hospitalizations, and even deaths, which are otherwise hard to compare due to differences in reporting systems; by relating the number of doctor consultations, hospitalizations and deaths to the serological attack rate we can assess country-specific biases in the reporting systems.

Although the pandemic strain is antigenically distinct from other currently circulating seasonal human influenza strains [20], reliable serological assays still need to be developed. Current standard techniques that have been used to quantify antigenic distance between strains depend on antibodies raised in animal models [21]. However, it is reasonable to expect that unpaired assays using human sera will give a good indication of prior exposure to the pandemic strain in most age groups [22], especially once cross-sectional data have been calibrated using paired sera. Despite these potential issues, it seems reasonable to assume that unpaired serological surveys will give an informative snap-shot of exposure history at a population-level and that it will be straightforward to characterize the degree of uncertainty associated with any single titration.

Pharmaceutical interventions will likely play little role in middle and low income countries.  Both the epidemiology and options for interventions are clearly different for less-developed countries compared with highly industrialized countries. Population density, mobility, household structure and school attendance patterns all differ significantly between and within regions. Therefore, it is not safe to assume that patterns of infection well-described in one population will be widely representative of the world’s population. In particular, after the initial Northern and Southern Hemisphere waves of infection, it will not be wise to assume that all other populations will experience similar infection attack rates. Empirical studies should be conducted in multiple representative populations. 

Building on existing demographic surveillance or influenza surveillance systems provides an option for many countries. Where possible, samples can be obtained from cross-sectional serological surveys. Where available, samples from national blood supply systems can provide real-time monitoring of infection incidence or cross reactive antibody responses to the H1N1pdm, as can residual blood samples taken from patients for diagnostic laboratory testing (although these samples will not necessarily represent the entire population). In low resource countries without such systems in place, surveillance systems for diseases such as dengue and polio could be adapted for H1N1pdm.  For example, in several countries in South East Asia, Central and South America, community-based surveillance studies were established to assess the burden of dengue among children and adults. Similar surveys have proved extremely useful during massive outbreaks of chikungunya in the Indian Ocean in 2006-2007 [23] [24]. Polio surveillance, which aims to identify all acute flaccid paralysis cases among children through reporting and laboratory testing, has wide geographic coverage in Africa and Asia. In addition, such systems, which routinely collect blood, could be used to evaluate antibody levels.  Lastly, seroprevalence studies that are currently planned or underway for HPAI/H5N1 in several African and Asian countries could also test for anti-H1N1pdm antibodies.

## Acknowledgements and Funding Information

The authors acknowledge support from the Bill and Melinda Gates Foundation (MVK, NMF, CF); Royal Society (CF); Medical Research Council (MVK, NMF, CF, PW); EU FP7 programme (NMF); UK Health Protection Agency (PW); US National Institutes of Health Models of Infectious Disease Agent Study program through cooperative agreement 1U54GM088588 (ML); NIH Director's Pioneer Award, DP1-OD000490-01 (DS); EU FP7 grant EMPERIE 223498 (DS); the Wellcome Trust (DS); R01 TW008246-01 from Fogerty International Center (SR); the RAPIDD program from Fogerty International Center with the Science & Technology Directorate, Department of Homeland Security (SR); the Research Fund for the Control of Infectious Disease, Hong Kong (SR); and the Institut de Veille Sanitaire Sanitaire funded by French Ministry of Health (JCD).

## Competing Interests

Peter White is a member of the UK government's Scientific Advisory Group for Emergencies (SAGE) and Scientific Pandemic Influenza Advisory Committee (SPI) and its modelling sub-group (SPI-M). He is employed by the UK Health Protection Agency. Marc Lipsitch has received consulting fees from the Avian/Pandemic Flu Registry (Outcome Sciences), which is funded in part by Roche. All other authors report no competing interests. 
